# Retraction Note: International trade and environmental pollution in sub-Saharan Africa: do exports and imports matter?

**DOI:** 10.1007/s11356-024-33137-9

**Published:** 2024-04-02

**Authors:** Emmanuel Duodu, Desmond Mbe-Nyire Mpuure

**Affiliations:** Department of Fundamentals of Economic Analysis, University of Alicante, Sant Vicent del Raspeig, Spain

**Retraction Note: Environmental Science and Pollution Research (2023) 30:53204-53220**

10.1007/s11356-023-26086-2

The Publisher has retracted this article in agreement with the Editor-in-Chief. An investigation by the Publisher found a number of articles, including this one, with a number of concerns, including but not limited to compromised peer-review process, inappropriate or irrelevant references, containing nonstandard phrases or not being in scope of the journal. Based on the investigation's findings the Publisher, in consultation with the Editor-in-Chief, therefore no longer has confidence in the results and conclusions of this article.

The authors have not stated whether they agree or disagree to this retraction.

